# Multiple non-cell-autonomous defects underlie neocortical callosal dysgenesis in *Nfib*-deficient mice

**DOI:** 10.1186/1749-8104-4-43

**Published:** 2009-12-04

**Authors:** Michael Piper, Randal X Moldrich, Charlotta Lindwall, Erica Little, Guy Barry, Sharon Mason, Nana Sunn, Nyoman Dana Kurniawan, Richard M Gronostajski, Linda J Richards

**Affiliations:** 1 Queensland Brain Institute, The University of Queensland, Brisbane, Queensland, Australia; 2 Institute for Neuroscience and Physiology, Sahlgrenska Academy, University of Gothenburg, Sweden; 3 Centre for Magnetic Resonance, The University of Queensland, Brisbane, Queensland, Australia; 4 Department of Biochemistry and the Program in Neuroscience, State University of New York at Buffalo, Buffalo, NY, USA; 5 Center of Excellence in Bioinformatics and Life Sciences, Buffalo, NY, USA; 6 The School of Biomedical Sciences, The University of Queensland, Brisbane, Queensland, Australia

## Abstract

**Background:**

Agenesis of the corpus callosum is associated with many human developmental syndromes. Key mechanisms regulating callosal formation include the guidance of axons arising from pioneering neurons in the cingulate cortex and the development of cortical midline glial populations, but their molecular regulation remains poorly characterised. Recent data have shown that mice lacking the transcription factor *Nfib *exhibit callosal agenesis, yet neocortical callosal neurons express only low levels of *Nfib*. Therefore, we investigate here how *Nfib *functions to regulate non-cell-autonomous mechanisms of callosal formation.

**Results:**

Our investigations confirmed a reduction in glial cells at the midline in *Nfib*^-/- ^mice. To determine how this occurs, we examined radial progenitors at the cortical midline and found that they were specified correctly in *Nfib *mutant mice, but did not differentiate into mature glia. Cellular proliferation and apoptosis occurred normally at the midline of *Nfib *mutant mice, indicating that the decrease in midline glia observed was due to deficits in differentiation rather than proliferation or apoptosis. Next we investigated the development of callosal pioneering axons in *Nfib*^-/- ^mice. Using retrograde tracer labelling, we found that *Nfib *is expressed in cingulate neurons and hence may regulate their development. In *Nfib*^-/- ^mice, neuropilin 1-positive axons fail to cross the midline and expression of neuropilin 1 is diminished. Tract tracing and immunohistochemistry further revealed that, in late gestation, a minor population of neocortical axons does cross the midline in *Nfib *mutants on a C57Bl/6J background, forming a rudimentary corpus callosum. Finally, the development of other forebrain commissures in *Nfib*-deficient mice is also aberrant.

**Conclusion:**

The formation of the corpus callosum is severely delayed in the absence of *Nfib*, despite *Nfib *not being highly expressed in neocortical callosal neurons. Our results indicate that in addition to regulating the development of midline glial populations, *Nfib *also regulates the expression of neuropilin 1 within the cingulate cortex. Collectively, these data indicate that defects in midline glia and cingulate cortex neurons are associated with the callosal dysgenesis seen in *Nfib*-deficient mice, and provide insight into how the development of these cellular populations is controlled at a molecular level.

## Background

Axonal fibre tracts enable the transfer of information between discrete parts of the brain. Within the cerebral cortex, the corpus callosum (CC), which comprises the largest fibre tract in the brain, provides connectivity between the left and right cerebral hemispheres [1,2]. The flow of information facilitated by this tract plays an integral role in many critical functions, including behaviour, emotion and higher order cognition. Indeed, defective development of this tract in humans is correlated with a large number of syndromes, such as Mowat Wilson syndrome and Aicardi syndrome, as well as disorders including autism and schizophrenia [3]. Formation of the CC requires a series of dynamic events to be co-ordinated both spatially and temporally during both embryogenesis and the postnatal period. These include correct patterning of the midline, differentiation and specification of callosal neurons within the nascent cortical plate, the development of distinct midline glial populations, targeting of axons to the contralateral hemisphere and the elimination of those supernumerary axons overproduced during development [1,4]. However, while the clinical significance of the CC has long been known, our understanding of the molecular determinants underlying formation of this fibre tract remains incomplete.

Research has begun to identify some of the molecular components regulating different aspects of callosal formation. For instance, the DNA-binding protein Satb2 was recently implicated as a key determinant controlling the specification of callosally projecting neurons within the cortex [5,6]. Furthermore, axon guidance cues, including Netrin 1 [7], class III semaphorins [8] and Slit2 [9], as well as guidance receptors such as DCC [10], neuropilin 1 (Npn1) [11], Robo1 [12] and Ryk [13] have been implicated in callosal development. In addition, the activity of callosal neurons during development is known to be required for axonal targeting and specificity through pruning within the contralateral hemisphere [4,14].

Another critical determinant of CC formation is the development of distinct glial populations at the cortical midline [15]. Two midline glial populations, the glial wedge and the indusium griseum glia, are believed to regulate callosal development, in part through expression of guidance cues such as Slit2 [16,17]. Given that these glia develop relatively early compared to other cortical glial populations [18], identifying the factors regulating their development is important for understanding both glial development and axonal guidance. One gene family in particular, the Nuclear Factor One (*Nfi*) transcription factors, has been shown to play a central role in regulating glial development and axon tract formation during embryogenesis. *Nfia *was recently implicated in regulating gliogenesis in the spinal cord [19], and *Nfia*-deficient mice have been demonstrated to have severely reduced glial formation at the cortical midline [20]. Similarly, *Nfib *has been shown to regulate the formation of glia within the ammonic neuroepithelium of the developing hippocampus [21].

Mice lacking *Nfib *have glial defects at the midline, as well as agenesis of the CC [22], but whether these defects are mechanistically related is unknown. Here we demonstrate that neither excessive apoptosis, nor aberrant proliferation at the midline, underlies the glial defect in *Nfib *knockout mice, but rather that radial progenitors fail to differentiate into mature glia. Furthermore, expression of *Slit2 *is diminished at the midline. We also report that cell-autonomous defects in cingulate cortex neurons may contribute to callosal malformation in *Nfib*^-/- ^mice. Finally, we demonstrate that, at embryonic day 18 (E18), a small population of axons does cross the midline caudally in *Nfib*-deficient mice. These results demonstrate that *Nfib *is critical for the maturation of midline glia that are required for CC formation. Furthermore, defects in Npn1-expressing pioneering neurons may contribute to the callosal dysgenesis evident in *Nfib*^-/- ^mice, implicating multiple non-cell-autonomous roles for *Nfib *in neocortical callosal formation.

## Results

### NFIB is expressed at the cortical midline

**Figure 1:**
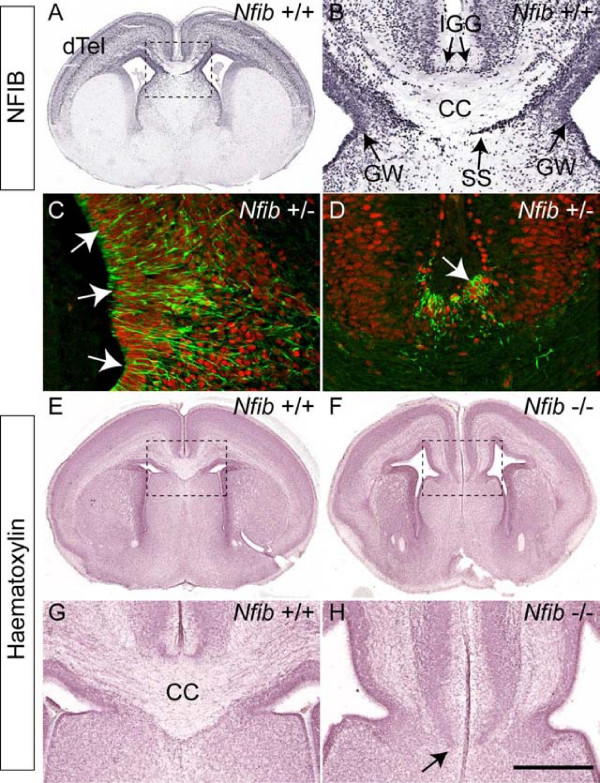
**Expression of NFIB in midline glial populations**. **(A, B) **Coronal section of an E18 wildtype brain stained with NFIB. (A) NFIB was expressed broadly throughout the dorsal telencephalon (dTel). (B) Higher magnification view of the boxed region in (A) showing NFIB expression at the cortical midline. NFIB was expressed in the glial wedge (GW), indusium griseum glia (IGG) and subcallosal sling (SS). **(C, D) **Confocal sections of E18 *Nfib *heterozygote brains, demonstrating the co-expression of the β-galactosidase (β-gal) reporter (red) and glial fibrillary acidic protein (GFAP; green) within the glial wedge (C) and the indusium griseum glia (D). β-gal-positive nuclei were often surrounded by GFAP-positive fibres (arrows in C, D), indicating that GFAP-expressing glia likely express *Nfib*. **(E-H) **Coronal sections of wildtype (E, G) and *Nfib *knockout (F, H) brains stained with haematoxylin. The corpus callosum (CC) does not form rostrally in mice lacking *Nfib *(arrow in (H)). Panels (G) and (H) are higher magnifications of the boxed regions in (E) and (F), respectively. Scale bars: 500 μm (A, E, F); 200 μm (B, G, H); 100 μm (C, D).

Although we previously showed by retrograde labelling that NFIB was not highly expressed in neocortical callosal neurons [23], we wanted to examine what other cells expressed NFIB at the midline. Immunohistochemistry on 6 μm coronal paraffin sections from E18 wildtype brains (Figure 1A) demonstrated the expression of NFIB within the glial wedge and the indusium griseum, two glial populations essential for development of the CC (Figure 1B). Expression was also detected in the subcallosal sling (Figure 1B), a stream of cells originating in the subventricular zone that crosses the cortical midline ventral to the developing CC [24,25]. The *Nfib *knockout line was generated with a nuclear-localised *lacZ *reporter gene [22]. Expression of β-galactosidase at the cortical midline in E18 heterozygote brains was co-incident with that of NFIB (Additional file 1), enabling us to use the expression of β-galactosidase as a reliable indicator of those cells expressing NFIB. To determine if the cells in the glial wedge and indusium griseum expressing *Nfib *were in fact glia, we double labelled E18 coronal sections with antibodies against β-galactosidase (a mouse monoclonal antibody) and glial fibrillary acidic protein (GFAP; a rabbit polyclonal antibody), a marker for mature glial cells. Many β-galactosidase-positive cells in both the glial wedge and the indusium griseum were surrounded by GFAP-positive fibres, indicating that these glial populations are likely to express *Nfib *(Figure 1C, D).

*Nfib*-deficient mice exhibit a variety of cortical deficits, including absence of the basilar pons [26] and malformation of the dentate gyrus [21]. They also exhibit agenesis of the CC at E17 as determined by staining with the axonal marker L1 [22]. Haematoxylin staining of E18 wildtype and *Nfib*-deficient brains (Figure 1E-H) supported this finding, indicating that, in rostral sections of the mutant, formation of the CC was impaired.

### Glial development is curtailed at the cortical midline of *Nfib*-deficient mice

**Figure 2:**
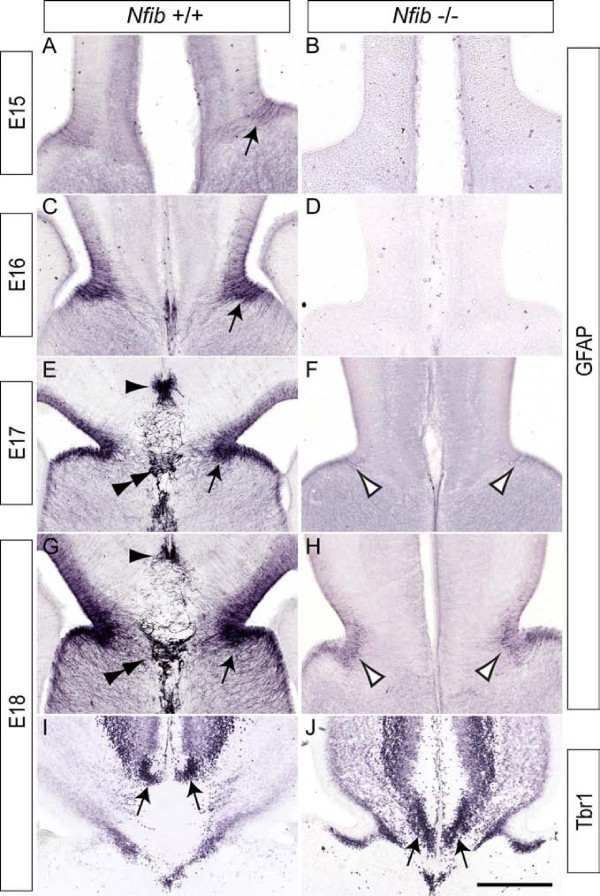
**Reduced expression of GFAP in *Nfib *knockout mice**. **(A-H) **Expression of GFAP at the cortical midline of wildtype (A, C, E, G) and *Nfib*-deficient (B, D, F, H) mice. In the wildtype, expression of GFAP in the glial wedge was initiated at E15, and became progressively stronger as development proceeded (arrows in (A, C, E, G)). GFAP expression in the indusium griseum glia (arrowhead) and midline zipper glia (double arrowhead) of the wildtype was also evident from E17 onwards (E, G). However, in the mutant, low levels of GFAP expression in the glial wedge were only observed from E17 onwards (open arrowheads in (F, H)), whereas expression in the indusium griseum glia and midline zipper glia was absent. Neurons in the indusium griseum of the wildtype expressed Tbr1 (arrows in (I)). Neurons expressing Tbr1 in the indusium griseum were also observed in the mutant (arrows in (J)). Scale bars: 280 μm (A, B); 250 μm (C, D); 225 μm (E, F); 200 μm (G-J).

The development of mature, GFAP-expressing glial populations at the cortical midline of *Nfib*^-/- ^mice at E17 was reported to be reduced in the absence of this transcription factor [22]. To gain a deeper insight into glial development during callosal formation, we first conducted a time-course of GFAP expression in *Nfib*-deficient and littermate control brains (Figure 2). In sections from wildtype mice, GFAP-expressing cells were first seen in the glial wedge at E15, and within the indusium griseum glia and a third midline glial population, the midline zipper glia, at E17 (Figure 2A, C, E). In the mutant, however, GFAP expression at the cortical midline was delayed until E17, when a small population of GFAP-expressing cells became evident within the glial wedge (Figure 2B, D, F). By E18, expression of GFAP within the glial wedge in the mutant had increased, but was still reduced in comparison to that seen in wildtype controls (Figure 2G, H). Moreover, at this rostro-caudal position, no GFAP expression was observed in the indusium griseum or in the midline zipper glia of the mutant mice. As the indusium griseum contains both glia and neurons [27], we also examined the expression of Tbr1, a marker of post-mitotic neurons. These data revealed that neurons were still present in the indusium griseum of mice lacking *Nfib *(Figure 2J), from which we infer that development of this structure *per se *is not affected. Rather, it is specifically the development of midline glial populations that is aberrant in the *Nfib *mutants.

### Proliferation and cell death is normal at the cortical midline of *Nfib *null mutants

**Figure 3:**
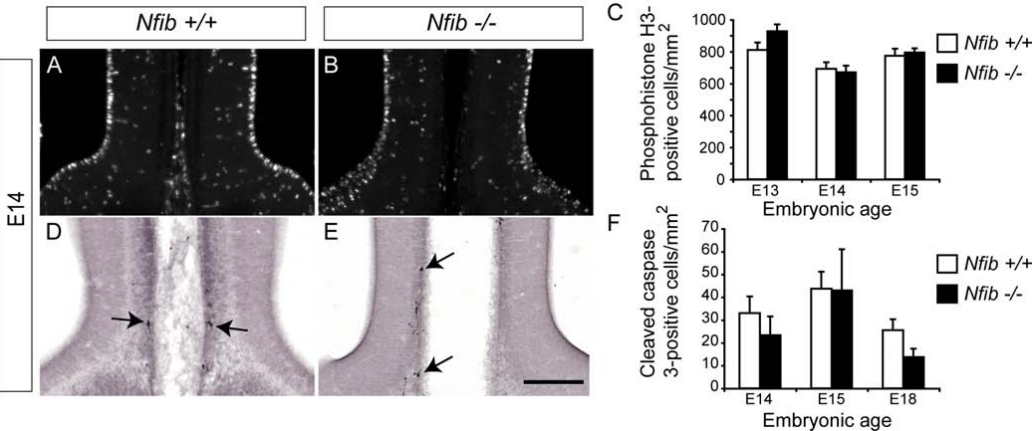
**Normal proliferation and cell death at the cortical midline of mice lacking *Nfib***. **(A, B) **Proliferation at the cortical midline in wildtype (A) and *Nfib*-deficient (B) mice was assessed with immunohistochemistry against the mitotic marker phosphohistone H3. **(C) **Counts of phosphohistone H3-positive cells at the cortical midline demonstrated that there was no significant difference in proliferation between *Nfib *null mutants and controls at E13, E14 or E15. **(D, E) **Apoptosis at the midline in wildtype (D) and *Nfib*-deficient (E) brains was assessed via expression of the marker for cell death, cleaved caspase 3. There were few apoptotic cells observed in either wildtype or knockout samples (arrows in (D, E)), and these were predominantly observed around the area where fusion between the cerebral hemispheres occurs. **(F) **We did not observe any significant differences in the numbers of apoptotic cells in mice lacking *Nfib *compared to wildtype controls at E14, E15 or E18. n = 3 independent replicates for both wildtype and *Nfib *mutants. Error bars indicate standard error of the mean. Scale bar: 300 μm.

The absence of cortical midline glial cells within *Nfib*-deficient mice may arise from deficiencies in cellular proliferation at the midline, excessive cellular apoptosis, or through a failure of cortical progenitors to differentiate into mature glia during embryogenesis. To determine which of these processes was responsible for the defect in glial development we observed in *Nfib *null mutants, we first investigated cellular proliferation at the cortical midline. We have demonstrated previously that glia within the glial wedge and indusium griseum are born predominantly between E13 and E15 [18]. We therefore analysed proliferation at the cortical midline at these ages in both wildtype and *Nfib*-deficient brains using the mitotic marker phosphohistone H3 (PH3; Figure 3A, B). As these data revealed no significant difference in the number of PH3-positive cells between *Nfib *mutant and wildtype sections at the cortical midline at E13, E14 or E15 (Figure 3C), we next investigated cell death using the apoptotic marker cleaved caspase 3. Very few cleaved caspase 3-positive cells were seen at the cortical midline of either wildtype or *Nfib*-deficient brains, and those that were found were located almost exclusively where the cerebral hemispheres fuse (arrows in Figure 3D, E). Furthermore, we observed no significant differences in apoptotic cell death at the midline between *Nfib *mutants and controls between E14 and E18 (Figure 3F). Collectively, these data indicate that neither aberrant proliferation nor excessive apoptosis are responsible for the phenotypic abnormalities observed in the absence of *Nfib*. We therefore next examined the specification of radial progenitor cells in *Nfib*-deficient brains, and their capacity to differentiate into mature glia.

### Radial progenitors are specified in the absence of *Nfib*

**Figure 4:**
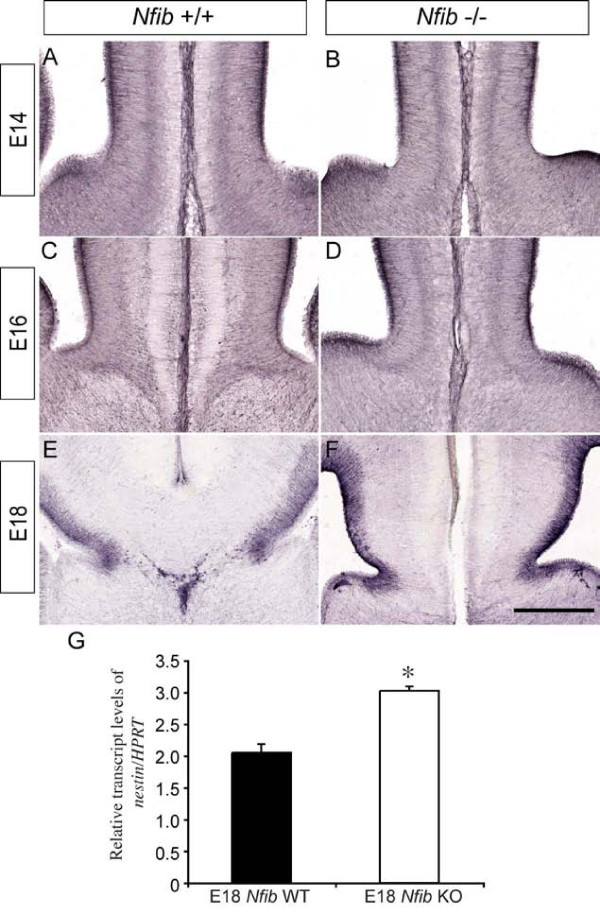
**Radial progenitor cells express *nestin *at higher levels at E18 in *Nfib *mutant mice**. **(A-F) **Coronal sections of wildtype (A, C, E) and *Nfib*-deficient brains (B, D, F) demonstrating expression of nestin. At E14 (A, B), E16 (C, D) and E18 (E, F), expression of nestin in the mutant was comparable to that in the control. **(G) **At E18, levels of *nestin *mRNA were significantly higher in *Nfib *mutants than in littermate controls (*P < 0.05; *t*-test). RNA from three independent replicates for both wildtype (WT) and *Nfib *mutants (*Nfib *knockout (KO)) was analysed. Error bars indicate standard error of the mean. Scale bar: 300 μm (A, B); 250 μm (C, D); 200 μm (E, F).

The intermediate filament protein nestin is expressed from E10.5 onwards in radial progenitors within the developing cortical neuroepithelium [28], and as such, nestin expression can be used to monitor the specification of radial progenitors within the cortex during embryogenesis [29]. Nestin expression at the cortical midline of *Nfib*-deficient mice between E14 and E18 was grossly normal when compared to that of littermate controls (Figure 4). However, analysis of *nestin *mRNA at E18 using quantitative real-time PCR (qPCR) on wildtype and *Nfib*-deficient cortical tissue showed that there were significantly higher levels of *nestin *in the *Nfib *mutant compared to wildtype controls at this age (*P *< 0.05, *t*-test; Figure 4G), indicative of a retention of progenitor cells in the mutant. These findings suggest that radial progenitors are specified in the absence of this transcription factor, yet are delayed in their differentiation to GFAP-positive glia in late gestation.

### Expression of radial glial markers at the midline is impaired

**Figure 5:**
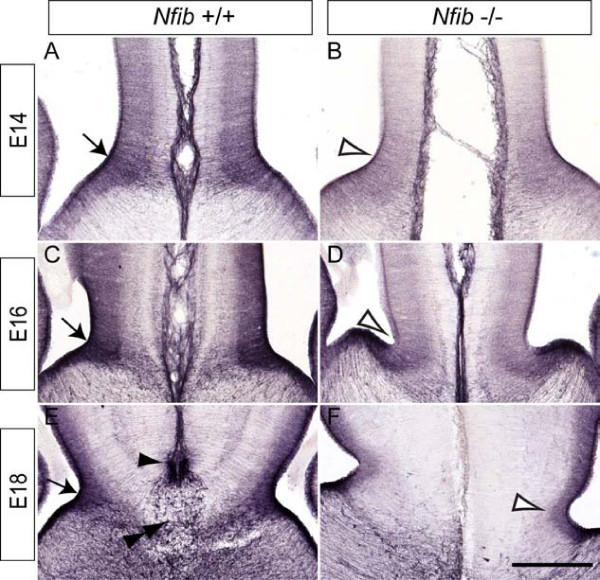
**Diminished expression of GLAST at the cortical midline of *Nfib*-deficient mice**. **(A-F) **Expression of GLAST at E14 (A, B), E16 (C, D) and E18 (E, F) in coronal sections of wildtype (A, C, E) and *Nfib *knockout (B, D, F) brains. At E14 in the wildtype (A), GLAST was expressed in the glial wedge (arrow), and this expression intensified as development proceeded (arrows in (C, E)). Furthermore, GLAST expression was observed in the indusium griseum glia (arrowhead) and midline zipper glia (double arrowhead) at E18 in the wildtype (E). In the mutant, however, GLAST expression in the glial wedge was reduced in comparison to controls (open arrowheads; compare (B) to (A), and (D) to (C)). Moreover, the indusium griseum glia and midline zipper glia in the *Nfib *null mutant were not apparent in the mutant via GLAST immunohistochemistry at E18 (F). Scale bars: 300 μm (A, B); 250 μm (C, D); 200 μm (E, F).

As nestin-expressing radial progenitors differentiate into radial glia, they begin expressing astroglial markers. Two glial-specific markers used previously to investigate the differentiation of radial progenitors in the cortex are astrocyte-specific glutamate transporter (GLAST) [30] and the extracellular matrix molecule tenascin C [31]. To determine whether the lack of GFAP-positive midline glia in *Nfib*-deficient mice was due to a defect in the differentiation of radial progenitors, we analysed the expression of these markers during development. At E14 in wildtype mice, GLAST expression was observed in the ventricular zone of the cortex, but was higher within the region of the presumptive glial wedge (Figure 5A). This expression pattern was even more evident in the wild type at E16 (Figure 5C). However, in mutant mice, expression of GLAST was clearly diminished in comparison to wildtype controls at both E14 and E16 (Figure 5B, D). At E18 in wildtype mice, GLAST expression was observed in the glial wedge, the indusium griseum glia and the midline zipper glia (Figure 5E). In mutant mice, GLAST immunoreactivity in the glial wedge was diminished, and moreover, this marker, like GFAP (Figure 2), was detected in neither the indusium griseum glia nor the midline zipper glia (Figure 5F), indicating that these mature glial populations appear absent in *Nfib*-deficient mice. Expression of tenascin C was also compromised in mice lacking *Nfib*. Comparison of wildtype and *Nfib*-deficient brains at E15 and E16 indicated that levels of this extracellular matrix molecule were higher in the wildtype than in the mutant (Additional file 2). Taken together, these data indicate that glial development is indeed impaired in *Nfib *knockout mice, and suggest that the lack of mature, GFAP-expressing glia at the cortical midline of the mutant results from aberrant differentiation of radial progenitors.

### Abnormal development of the subcallosal sling in *Nfib *knockout mice

**Figure 6:**
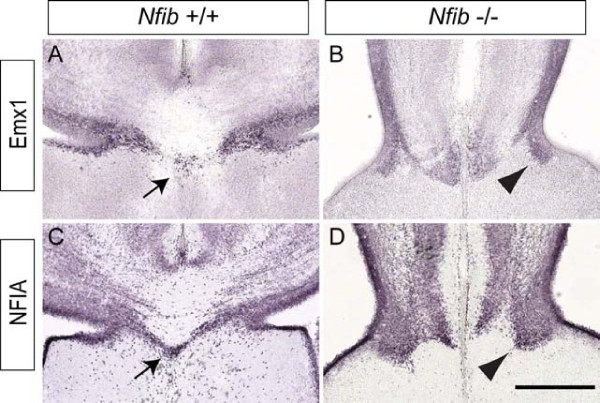
**The subcallosal sling fails to form correctly in *Nfib *knockout mice**. **(A-D) **Coronal sections of E18 brains showing expression of Emx1 (A, B) and NFIA (C, D). In the wildtype, cells of the subcallosal sling were seen crossing the midline immediately ventral to the CC (arrows in (A, C)). In the mutant, however, cells of the sling did not cross the midline, and instead remained ipsilateral (arrowheads in (B, D)). Scale bar: 200 μm.

The subcallosal sling is a population of neurons that migrate from the medial aspect of the lateral ventricles beneath the nascent CC. Migration of these neurons begins at approximately E15 and continues postnatally [24]. To determine whether formation of this structure was perturbed in mice lacking *Nfib*, we performed immunohistochemistry using two markers expressed by sling neurons, NFIA and Emx1 [24]. In wildtype mice at E18, sling neurons were observed beneath the CC (Figure 6A, C). In *Nfib *mutant mice, however, sling neurons were unable to cross the midline, perhaps due to the failure of the CC to form. Unlike in *Nfia*-deficient mice, where sling cells invade the septum [20], these cells appear to stall near the midline in the absence of *Nfib *and do not invade the septum (Figure 6B, D). This phenotype was also seen with a third marker for the subcallosal sling, calretinin (data not shown). These data suggest that correct formation of the sling requires *Nfib *function, and further indicates that *Nfia *and *Nfib *may play different roles in relation to subcallosal sling formation.

### Expression of Npn1 on the axons of cingulate pioneer neurons is diminished in *Nfib*-deficient mice

**Figure 7:**
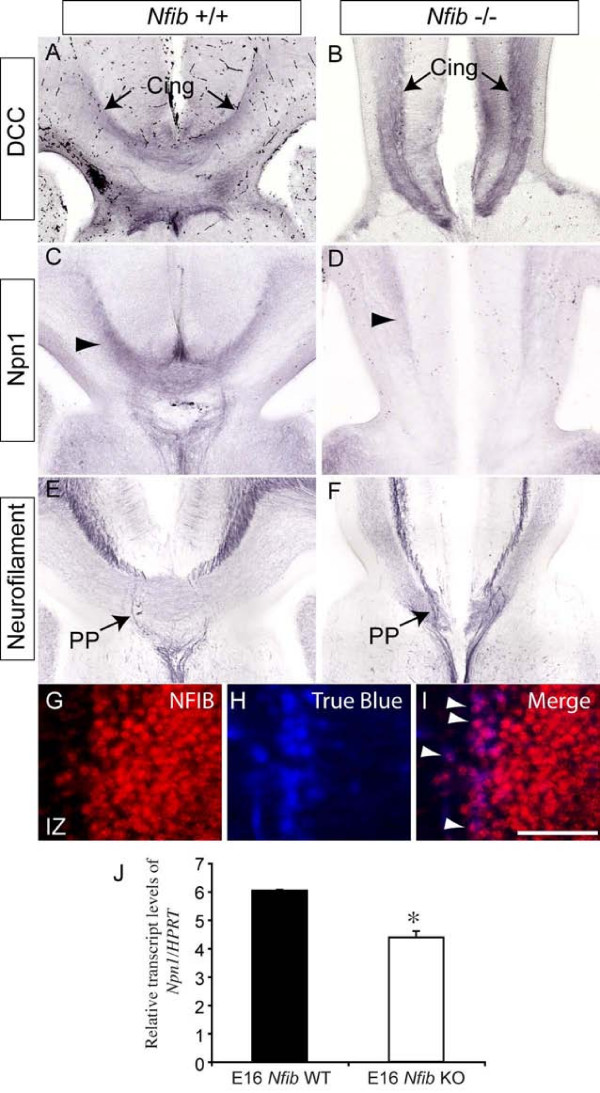
**Expression of guidance receptors on the axons of cingulate pioneering neurons**. **(A-F) **Expression of DCC (A, B), Npn1 (C, D) and neurofilament (E, F) in coronal sections of E18 wildtype (A, C, E) and *Nfib*-deficient (B, D, F) brains. Axons from neurons in the cingulate cortex initiate callosal tract formation, and express the guidance receptor DCC. Expression was seen on axons from the cingulate cortex (Cing) in both the wildtype and *Nfib *null mutant (arrows in (A, B)). However, expression of Npn1, another guidance receptor localised to cingulate pioneering axons, was diminished in the knockout in comparison to littermate controls (arrowheads in (C, D)). The perforating pathway (PP), shown via expression of the axonal marker neurofilament in wildtype sections (arrow in (E)), appeared relatively normal in mice lacking *Nfib *(arrow in (F)). **(G-I) **The retrograde tracer True Blue was injected into the cingulate cortex of E17 wildtype embryos *in utero*. At E18, immunohistochemistry on coronal sections against NFIB (G) demonstrated that the retrograde tracer (H) and NFIB were co-localised in a population of callosally projecting neurons in the cingulate cortex (arrowheads in (I)). **(J) **At E16, levels of *Npn1 *mRNA were significantly lower in *Nfib *mutants than in littermate controls (*P < 0.05; *t*-test). RNA from three independent replicates for both wild type (WT) and *Nfib *mutants (*Nfib *knockout (KO)) was quantified. Error bars indicate standard error of the mean. IZ, intermediate zone. Scale bar: A-F 200 μm; G-I 30 μm.

The failure of the glial wedge and the indusium griseum glia to form may underlie the callosal defects previously described for *Nfib*-deficient mice [22], as these glial populations have been postulated to be pivotal for the formation of this axonal tract [16,17]. We next investigated whether defects in callosally projecting neurons could contribute to the CC defects observed in *Nfib*^-/- ^mice. Two populations of neurons extend axons that cross the midline via the CC; neocortical callosal neurons and cingulate cortex callosal neurons [1]. The latter are thought to be critical for pioneering callosal tract formation, as neurons from the cingulate cortex are the first to extend axons across the CC [32,33]. Interestingly, retrograde labelling in early postnatal (P5) wildtype mice has previously shown that most callosally projecting neocortical neurons do not express NFIB at this age [23]. However, the broad expression pattern of NFIB in the cingulate cortex at E18 (Figure 1A) suggests that, in addition to glial deficits, cell-autonomous neuronal defects within the cingulate cortex may contribute to the callosal phenotype in embryonic *Nfib*-deficient mice. To investigate this, we analysed projections arising from the cingulate cortex. Deleted in colorectal cancer (DCC) is a marker expressed selectively on callosal axons arising from neurons within the cingulate cortex [34]. In wild types at E18, DCC-expressing axons could be seen crossing the midline in the dorsal region of the CC (Figure 7A). In the mutant, DCC-expressing axons extended towards the midline (Figure 7B) but failed to cross. These data indicate that neurons within the cingulate cortex were indeed able to extend axons towards the presumptive CC, and that this was not a growth defect. However, expression of another guidance receptor specifically localised to cingulate cortex axons, Npn1 [8], was diminished in *Nfib*-deficient mice in comparison to wildtype controls (Figure 7C, D). Levels of *Npn1 *mRNA were also significantly lower in the cortex of *Nfib *mutants at E16, the age at which Npn1-expressing axons from cingulate pioneer neurons initiate CC formation (*P *< 0.05, *t*-test; Figure 7J).

We have recently postulated that Npn1-semaphorin signalling may be vital for callosal formation [8]. Thus, diminished expression of Npn1 on axons emanating from the cingulate cortex could also contribute to the callosal defects observed in *Nfib *knockout mice. To determine if NFIB was expressed in cingulate cortex neurons extending axons across the CC, we performed tract tracing using the retrograde tracer True Blue, which was injected into the cingulate cortex of E17 embryos *in utero*. Embryos were then perfuse-fixed at E18. Immunohistochemistry against NFIB demonstrated that some nuclei in the cingulate cortex contralateral to the injection site were both NFIB- and True Blue-positive (Figure 7G-I), indicating that a proportion of callosally projecting cingulate neurons express *Nfib*. Interestingly, however, not all of the projections from the cingulate cortex were abnormal in the absence of *Nfib*. The perforating pathway is an ipsilateral tract extending from the cingulate cortex to the medial septum/diagonal band of Broca and *vice versa *[35]. Analysis of this pathway with immunohistochemistry against the axonal marker neurofilament revealed no defect, as in both wildtype and *Nfib*-deficient sections, neurofilament-positive axons comprising the perforating pathway intersected the callosal axons *en route *to their targets (Figure 7E, F).

### *Nfib*-deficient mice exhibit callosal dysgenesis at E18

**Figure 8:**
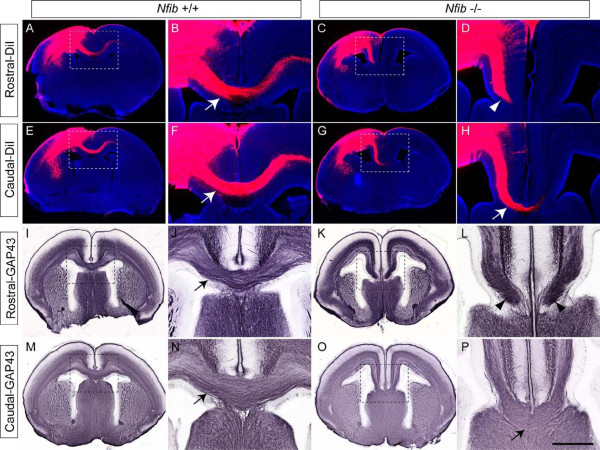
***Nfib*-deficient mice exhibit callosal dysgenesis**. **(A-H) **Carbocyanine tract tracing in wildtype (A, B, E, F) and *Nfib *mutant (C, D, G, H) brains at E18. DiI was injected into the neocortex of wildtype and knockout brains, thereby labelling all neocortical projections, including the CC. In the wildtype at rostral (A, B) and caudal (E, F) levels, callosal axons were seen projecting across the midline (arrows in (B, F)). In the knockout at rostral levels, no axons were observed crossing the midline (arrowhead in (D)). However, at more caudal levels, a small number of axons were seen crossing into the contralateral cortex (arrow in (H)). **(I-P) **Immunohistochemistry against the axonal marker GAP43 in wildtype (I, J, M, N) and *Nfib *mutant (K, L, O, P) brains at E18. The CC was clearly observed in the wildtype at rostral and caudal levels (arrows in (J, N)). In the mutant at rostral levels, no GAP43-positive axons were seen crossing the midline. Instead, axons stopped adjacent to the midline (arrowheads in (L)). More caudally, however, a small CC was evident in the mutant (arrow in (P)). Panels (B, D, F, H, J, L, N, P) are higher magnifications of the boxed regions in (A, C, E, G, I, K, M, O), respectively. Scale bars: 500 μm (A, C, E, G, I, K, M, O); 200 μm (B, D, F, H, J, L, N, P).

*Nfib*-deficient mice have been reported to exhibit callosal agenesis at E17, as axons expressing the cell adhesion molecule L1 reach, but do not cross, the midline in the absence of *Nfib *[22]. L1 is a non-specific marker for axons, and labels axons from both the cingulate cortex and neocortex. As our DCC labelling had indicated that cingulate cortex axons extend towards the cortical midline (Figure 7B), it remained a possibility that the L1-positive axons previously reported at the midline [22] arose not from the neocortex, but from the cingulate cortex. To investigate the origin of axons arriving at the midline, we performed tract tracing by injecting the carbocyanine dye DiI into the neocortex of both wildtype and *Nfib*-deficient mice at E18. Coronal sections at rostral and caudal levels in the wild type indicated that DiI-labelled axons were coursing through the CC and ascending into the contralateral hemisphere (Figure 8A, B, E, F). At rostral levels in the mutant, DiI-positive axons could be seen approaching, but not crossing, the cortical midline (Figure 8C, D). Furthermore, at E18 most callosally projecting neocortical axons located in layers II/III and V, identified by the expression of Satb2 [5], did not express NFIB (Additional file 3). These data indicate that, in the absence of *Nfib*, neocortical callosal axons are able to reach the midline, implying that callosal malformation in *Nfib *mutants does not arise from cell-autonomous defects within neocortical neurons. Unexpectedly, at more caudal levels, we observed a small population of axons crossing the cortical midline in the *Nfib *mutants (Figure 8G, H), indicating that, at this age, *Nfib*-deficient mice exhibit dysgenesis, not agenesis, of the CC. These findings were supported by analysis of the axonal marker GAP43, which indicated that there is indeed a population of axons that cross the midline via the CC at caudal levels in mice lacking *Nfib *(Figure 8I-P).

### Reduced expression of *Slit2 *at the cortical midline of *Nfib *mutants

**Figure 9:**
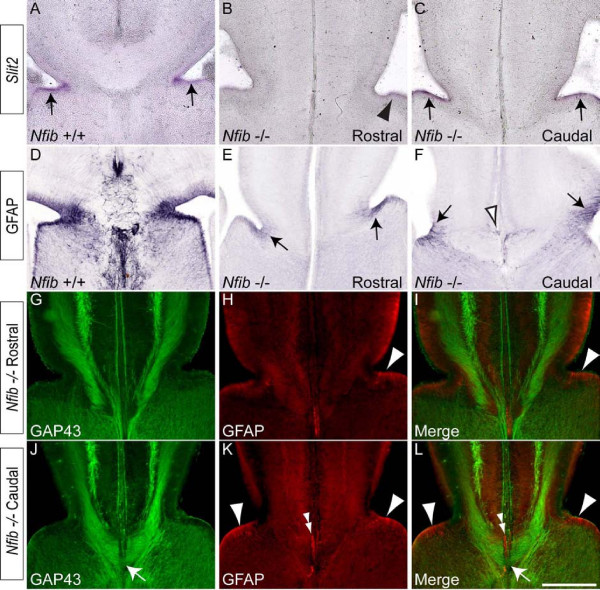
***Slit2 *expression at the cortical midline**. **(A) **In the wildtype at E18, expression of the axon guidance cue *Slit2 *was observed within the glial wedge region (arrows in (A)). **(B) **In the *Nfib *null mutant at rostral levels, expression of *Slit2 *was diminished (arrowhead). **(C) **At more caudal levels in the mutant, expression of *Slit2 *in the glial wedge region was more noticeable (arrows). **(D) **Expression of GFAP in the wildtype at E18. **(E) **In *Nfib *knockout sections at rostral levels, GFAP was observed in the glial wedge (arrows). **(F) **Further caudally, GFAP was observed in both the glial wedge (arrows) and indusium griseum glia (open arrowhead). **(G-L) **Co-immunofluorescent labelling of *Nfib *knockout sections at rostral (G-I) and caudal (J-L) levels with the axonal marker GAP43 (green) and the astrocytic marker GFAP (red). At rostral levels, no callosal axons could be seen crossing the midline, and few GFAP-positive glia were observed within the glial wedge (arrowheads in (H, I)). At caudal levels, however, more GFAP-positive glia were detected within the glial wedge (arrowheads in (K, L)) and GFAP-positive glia were also seen within the indusium griseum (double arrowheads in (K, L)). Callosal axons are also seen crossing the midline at this level (arrows in (J, L)). Scale bar: 200 μm.

Mice lacking the guidance molecule *Slit2 *exhibit callosal defects [9], and expression of *Slit2 *is also reduced within the glial wedge of *Nfia *mutants [20]. Given the dysgenic phenotype of *Nfib*-deficient mice, we next examined the expression of *Slit2 *to determine whether the phenotype we observed could be related to altered expression of this molecule. At E18, expression of *Slit2 *mRNA in the wildtype was observed within the glial wedge (Figure 9A). At rostral sections in the mutant, however, *Slit2 *expression was markedly lower (Figure 9B), whereas at more caudal levels it was at a level more comparable to that seen in controls (Figure 9C), which could contribute to the formation of the CC caudally in the mutant. Next, we analysed expression of GFAP more caudally in *Nfib*-deficient mice, to determine whether there were more mature glia within the region in which the callosal axons cross the midline. At rostral levels, few GFAP-positive glia were detected within the glial wedge region (Figure 9E). In more caudal regions, however, more GFAP-positive fibres were observed within the glial wedge, and GFAP-expressing cells were also found within the indusium griseum (Figure 9F). Finally, we performed co-immunofluorescent labelling of E18 *Nfib*^-/- ^brains with both GAP43 and GFAP. At rostral levels in the mutant, GFAP-positive glia were only observed within the glial wedge, and no axons were seen crossing the midline (Figure 9G-I). More caudally, however, labelling revealed GFAP-positive glia within both the glial wedge and indusium griseum, and callosal axons were observed crossing the midline (Figure 9J-L). Collectively, these data suggest that callosal formation in caudal regions of the *Nfib *mutant may be facilitated by the development, albeit delayed, of glial populations.

### Defects in multiple forebrain commissures in *Nfib *mutants

**Figure 10:**
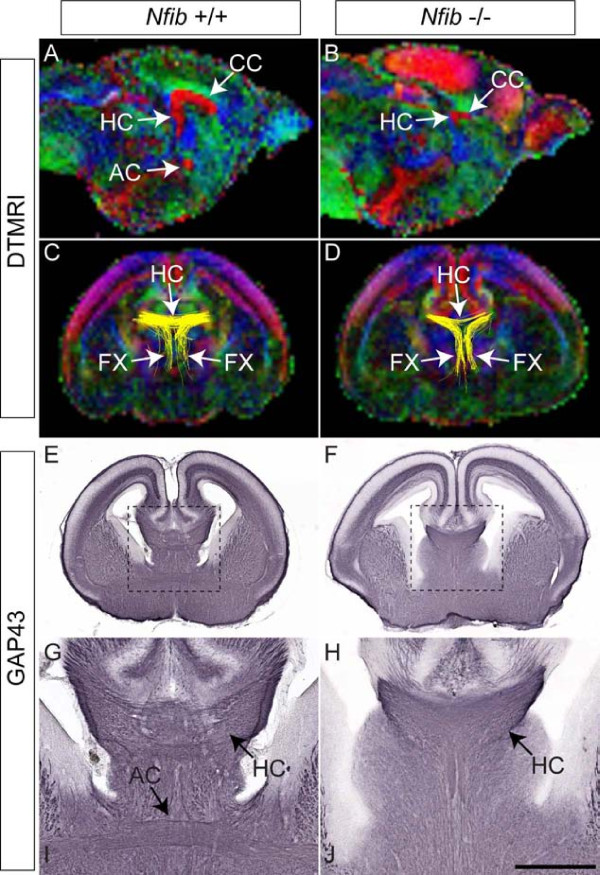
**Hippocampal commissure formation in *Nfib *null mutants**. **(A-D) **Colour-coded anisotropy maps of E18 wildtype (A, C) and *Nfib*-deficient (B, D) brains. The colour code indicates the direction of axon fibre tracts (blue, dorso-ventrally projecting tracts; red, medio-laterally projecting tracts; green, rostro-caudally projecting tracts). Sections in (A, B) are mid-sagittal views. In the wildtype (A), the three major forebrain commissures were evident; the corpus callosum (CC), the hippocampal commissure (HC) and the anterior commissure (AC). In the *Nfib *mutant, the anterior commissure was absent, but the corpus callosum and the hippocampal commissure were evident, although much reduced in size. (C, D) Coronal views of the brains scanned in (A, B), in which tractography (yellow lines) was performed on the hippocampal fimbria. The tracts to the hippocampal commissure and the fornix (FX) could be seen at this rostro-caudal position. In the *Nfib*-deficient brain (D), the size of the hippocampal commissure was reduced in comparison to that of the wildtype control (C). **(E-H) **The brains represented in (A, B) were cut coronally and axon tracts were revealed via expression of the axonal marker GAP43. In the wildtype (E, G), the hippocampal commissure and anterior commissure were seen crossing the midline. In the *Nfib *mutant (F, H), a reduced hippocampal commissure was revealed by GAP43 immunohistochemistry, and the anterior commissure was absent. Panels (G, H) are higher magnifications of the boxed regions in (E, F), respectively. Scale bars: 800 μm (A, B); 500 μm (C-F); 200 μm (G, H).

How the lack of *Nfib *affects the development of other forebrain commissures such as the hippocampal commissure and the anterior commissure is unknown. To address forebrain commissure development in *Nfib*-deficient mice, we performed diffusion tensor magnetic resonance imaging (DTMRI) to visualise the major axon tracts within the brain [36]. The axon tracts identified by DTMRI were colour-coded to denote the direction in which the fibre bundles project (blue, dorso-ventrally; red, medio-laterally; green, rostro-caudally). In a sagittal view of the midline of an E18 wildtype brain, all three major forebrain commissures (CC, hippocampal commissure and anterior commissure) were evident (Figure 10A). In the mutant, however, the anterior commissure was absent, and the CC and the hippocampal commissure, although evident, were reduced in comparison to that of the control (Figure 10B). This result was confirmed using tractography, which demonstrated a marked reduction in axon bundles within the hippocampal commissure of *Nfib*-deficient mice (Figure 10C, D). Furthermore, conventional analysis of these scanned brains using immunohistochemistry against GAP43 indicated the absence of the anterior commissure in the mutant (Figure 10E-H).

## Discussion

The *Nfi *gene family is required for multiple aspects of central nervous system development [37]. Here we show that *Nfib *regulates the differentiation of specific glial populations critical for formation of the CC, the glial wedge and the indusium griseum glia. In the absence of *Nfib*, the appearance of these glial populations is dramatically curtailed, although this is not due to aberrant apoptosis or proliferation. Rather, differentiation of these glia from radial progenitors at the cortical midline is impaired. Our data also indicate that expression of Npn1 on axons of pioneer neurons within the cingulate cortex of *Nfib *mutants is significantly reduced in comparison to controls. Collectively, these findings demonstrate that predominantly non-cell-autonomous defects contribute to the defects in neocortical CC formation in *Nfib*-deficient mice. Finally, we also show that, in late gestation, a small population of axons crosses the cortical midline at more caudal levels in *Nfib *mutant mice. This correlates with an increase in the levels of GFAP and *Slit2*, suggesting that, at least caudally, a compensatory mechanism allows both glial differentiation and CC formation in mice lacking this transcription factor.

The *Nfi *transcription factors are emerging as central players in glial lineage determination. *Nfi *genes have previously been shown to promote the expression of a suite of glial-specific genes *in vitro*, including those encoding GFAP [38], brain lipid binding protein [39], α_1_-antichymotrypsin [40] and tenascin C [41]. Furthermore, a number of recent studies have corroborated these findings *in vivo*. For instance, *Nfib *has been shown to regulate glial differentiation within the ammonic neuroepithelium of the developing mouse hippocampus [21], while the onset of gliogenesis in the chick spinal cord requires the action of *Nfia *[19]. A mechanistic insight into how *Nfi *genes could promote gliogenesis within cortical precursors has recently been proposed [42]. Namihira and colleagues [42] demonstrated that within cortical neural progenitor cells at mid-gestation, Notch pathway signalling elicited expression of NFIA, which bound to the promoters of astrocytic genes such as that encoding GFAP, culminating in demethylation and subsequent activation of these glial genes. Our findings are readily accommodated within the conceptual framework provided by these studies, with the impairment of glial maturation at the cortical midline being a direct consequence of radial progenitors failing to differentiate in the absence of *Nfib*.

Our data indicate that *Nfib *may also contribute to callosal formation via the cell-autonomous regulation of Npn1 within neurons of the cingulate cortex. Npn1, a cognate ligand for secreted class III semaphorins, has recently been shown to contribute to the formation of the CC [8,11,43]. The finding that Npn1 expression is significantly downregulated in the cortex of *Nfib *mutants implies that the role of *Nfi *genes is not solely confined to gliogenesis, a result that is supported by the broad expression pattern of this gene family within neurons and glia of the developing telencephalon [21,23]. Indeed, recent reports have highlighted significant roles for *Nfi *family members in neuronal development. For instance, *Nfib *has been shown to play a central role in precerebellar mossy fibre neuron generation within the pons [26], while *Nfia *controls axon outgrowth, dendritogenesis and migration of cerebellar granule neurons via regulation of N-cadherin and ephrin B1 [44].

Our finding that a rudimentary CC forms caudally in *Nfib*-deficient mice appears inconsistent with the initial characterisation of this knockout line, which reported agenesis of the CC [22]. This discrepancy could lie in the age of the embryos investigated, given Steele-Perkins and colleagues conducted their analysis at E17, one day prior to when the present analysis was conducted. Furthermore, the previous study analysed the *Nfib *allele on a mixed genetic background (129S6/C57Bl/6J). The 129S6 strain exhibits sporadic occurrence of callosal agenesis [45,46], which may have contributed to the complete absence of the CC observed [22]. The delayed development of the CC we described in this study is of considerable interest, as it raises the possibility that further development of this tract may occur postnatally. Unfortunately, on the genetic background on which this strain is maintained (C57Bl/6J), *Nfib *knockout mice die at birth due to defective lung maturation [22,47]. Generation of a conditional *Nfib *allele would be an ideal way to ablate *Nfib *in a cortex-specific manner, thereby enabling further investigation of CC formation postnatally in the absence of this transcription factor. Furthermore, the functional sequelae arising from delayed callosal malformation in these mice could then be investigated using behavioural analyses.

While a hypomorphic CC does form caudally at E18 in the absence of *Nfib*, how this is regulated remains unclear. One possibility is that the caudal CC axons in the *Nfib *mutant could utilise axons of the hippocampal commissure as a substrate to cross the cortical midline [48]. Another possible determinant of CC formation in the mutant is the delayed development of midline glia within both the glial wedge and indusium griseum. Although the identities of the genes regulating the delayed glial development in the E18 mutant are unknown, other members of the *Nfi *gene family are excellent candidates. *Nfia *and *Nfix *are both expressed within the glial wedge and indusium griseum at late gestation [23], and furthermore, *Nfia *and *Nfib *are expressed in the same cells within the glial wedge at E18 (data not shown). Thus, although differentiation of radial progenitors is delayed in the absence of *Nfib*, compensation by other *Nfi *family members may provide a mechanism for mature glia to eventually form at the cortical midline, thereby enabling development of the CC. Finally, our finding that the anterior commissure and the hippocampal commissure were also disrupted in the *Nfib*^-/- ^mice could indicate that similar non-cell-autonomous mechanisms, such as the development of midline glial and neuronal sling populations, may underlie the formation of all telencephalic commissures. In conclusion, our data provide a comprehensive insight into the phenotypic abnormalities underlying callosal malformation in *Nfib*-deficient mice, and demonstrate that multiple factors contribute to these defects during embryogenesis.

## Materials and methods

### Animals

Litters of wildtype C57Bl/6J and *Nfib*-deficient mice, bred at The University of Queensland with approval from the institutional Animal Ethics Committee, were used in this study. The *Nfib*^-/- ^allele [22] was backcrossed for more than ten generations onto the C57Bl/6J background. *Nfib*^+/- ^mice were bred to obtain wildtype, *Nfib*^+/- ^and *Nfib*^-/-^progeny. No midline defects were detected in wildtype or heterozygote animals. Timed-pregnant females were obtained by placing male and female mice together overnight. The following day was designated as E0 if the female had a vaginal plug. Embryos were genotyped by PCR as previously described [22].

### Fixation

On the required gestational day, embryos were drop-fixed in 4% paraformaldehyde (PFA; E14 and below) or transcardially perfused with 0.9% phosphate buffered saline, followed by 4% PFA (E15 to E18). They were then postfixed in 4% PFA at 4°C until sectioning.

### Haematoxylin staining

Brains of E18 wildtype C57Bl/6J or *Nfib*^-/- ^embryos were dissected from the skull, blocked in 3% noble agar (Difco, Sparks, MD, USA), and then sectioned coronally at 45 μm on a vibratome (Leica, Nussloch, Germany). Sections were then mounted and stained with Mayer's haematoxylin using standard protocols.

### Immunohistochemistry on floating sections

Brains were sectioned as described above, then processed free-floating for immunohistochemistry using the chromogen 3,3' diaminobenzidine as described previously [49]. Primary antibodies used for immunohistochemistry were anti-GAP43 (mouse monoclonal, 1/100,000; Chemicon, Bedford, MA, USA), anti-GFAP (rabbit polyclonal, 1/50,000; DAKO, Glostrup, Denmark), anti-cleaved caspase 3 (rabbit polyclonal, 1/1,000; Cell Signaling Technology, Danvers, MA, USA), anti-GLAST (rabbit polyclonal, 1/50,000; a gift from Niels Danbolt, University of Oslo), anti-nestin (mouse monoclonal, 1/1,500; Developmental Studies Hybridoma Bank), anti-tenascin C (rabbit polyclonal, 1/2,000; Chemicon), anti-Tbr1 (rabbit polyclonal, 1/100,000; a gift from Robert Hevner, University of Washington), anti-NFIA (rabbit polyclonal, 1/30,000; Active Motif, Carlsbad, CA, USA), anti-Emx1 (rabbit polyclonal, 1/30,000; a gift from Giorgio Corte, The University of Genova Medical School), anti-Npn1 (rabbit polyclonal, 1/75,000; a gift from David Ginty, Johns Hopkins University) and anti-DCC (rabbit polyclonal, 1/30,000; a gift from Helen Cooper, Queensland Brain Institute). Secondary antibodies used were biotinylated goat-anti-rabbit IgG (1/1,000; Vector Laboratories, Burlingame, CA, USA) and biotinylated donkey-anti-mouse IgG (1/1,000; Jackson ImmunoResearch, West Grove, PA, USA). To perform immunofluorescent labelling, sections were incubated overnight with the primary antibody at 4°C. They were then washed and incubated in secondary antibody, before being washed again and mounted. The primary antibodies used for immunofluorescent labelling were anti-phosphohistone H3 (rabbit polyclonal, 1/1,000; Millipore, Billerica, MA, USA), anti β-galactosidase (1/1,000; Promega, Madison, WI, USA), anti-GAP43 (1/5,000), anti-DCC (1/1,000), anti-GFAP (1/2,000), anti-Satb2 (1/1,000; Abcam, Cambridge, UK) and anti-NFIB (1/1,000). The secondary antibodies used were goat-anti-rabbit IgG AlexaFluor488 and goat-anti-mouse IgG AlexaFluor594 (both 1/1,000; Invitrogen, Carlsbad, CA).

### Immunohistochemistry on paraffin sections

E18 wildtype brains were perfused as above and embedded in paraffin wax. Brains were sectioned at a thickness of 6 μm. Antigen retrieval was performed using a 10 mM, pH 6 sodium citrate solution, and immunohistochemistry was performed as described above using 3,3' diaminobenzidine as the chromogen. The primary antibody used for immunohistochemistry was anti-NFIB (1/1,000, Active Motif), and a biotinylated goat-anti-rabbit IgG secondary antibody (Vector Laboratories) was used at 1/1,000.

### Image acquisition and analysis

Following immunohistochemistry, sections were imaged using an upright microscope (Zeiss Z1, Zeiss, Goettingen, Germany) attached to a digital camera (Zeiss AxioCam HRc). AxioVision software (Zeiss) was used to capture images. When comparing wildtype to knockout tissue, sections from matching positions along the rostro-caudal axis were selected.

### Quantification of proliferation

To quantify proliferation at the developing cortical midline, sections from E13, E14 and E15 wildtype C57Bl/6J or *Nfib*^-/- ^embryos were labelled with an anti-phosphohistone H3 antibody as described above. Sections were imaged using an upright fluorescence microscope (Zeiss Z1) attached to a digital camera (Zeiss AxioCam HRc). Eight to ten optical sections encompassing the entire 45-μm section were captured with an ApoTome (Zeiss). To calculate the total number of phosphohistone H3-positive cells per unit area at the cortical midline, a 300 μm^2^-boxed region, encompassing the presumptive glial wedge area, was generated using AxioVision software (Zeiss). The number of immunolabelled cells in focus in each optical section of this region was counted and pooled (n = 3 for both wildtype and knockout at all ages).

### Statistical analysis

For all experiments described in this study, sections from three different brains of each genotype were analysed. Statistical analyses were performed using a two-tailed unpaired *t-*test. Error bars represent standard error of the mean.

### Carbocyanine tract tracing

E18 wildtype and *Nfib*^-/- ^brains were fixed in 4% PFA as described above. A small injection of DiI (in a 10% solution of dimethylformamide; Invitrogen) was then made into the neocortex using a pulled glass pipette attached to a picospritzer. Brains were stored in the dark at 37°C in 4% PFA for at least 4 weeks to allow dye transport. They were then sectioned coronally at 45 μm using a vibratome, and imaged using an upright fluorescence microscope (Zeiss Z1). Nuclei were counterstained with 4',6-diamidino-2-phenylindole (DAPI; blue). Three brains were analysed for each genotype.

### Retrograde labelling under ultrasound guidance

Pregnant mice were anaesthetised with isofluorane (2%) for the duration of the microinjection procedures. The uterine horn was exposed through an incision in the abdominal midline for the purpose of ultrasound- imaging and guided microinjections (Vevo770, VisualSonics, Toronto, Canada). Retrograde labelling of callosal axons with True Blue chloride (Invitrogen) was performed as described previously, with modifications appropriate for ultrasound-guided microinjection *in utero*. Embryos were visualised under a 40 MHz transducer probe (RMV711) and a small volume of the tracer (approximately 250 nL of a 1 μg/μL solution) was injected into the cortex of wildtype E17 embryos *in utero *through the uterine wall with the aid of a nanojector (Nanoject II, Drummond Scientific, Broomall, PA, USA). Once embryos were injected, the uterine horn was returned to the abdominal cavity, and the incision was sutured. Then, 24 hours later (E18) the embryos were perfused transcardially as described above and processed for NFIB immunohistochemistry. Fluorescence images were obtained with an upright microscope (Zeiss Z1) as described above.

### *In situ *hybridisation

*In situ *hybridisation was performed as described previously [50], with minor modifications. An antisense riboprobe specific to *Slit2 *was hybridised to coronal brain sections at 65°C overnight. The colour reaction solution was BM Purple (Roche, Mannheim, Germany).

### Reverse transcription and quantitative real-time PCR

The reverse transcription was performed using Superscript III (Invitrogen). Briefly, 0.5 μg total RNA was reverse transcribed with random hexamers. qPCR reactions were carried out in a Rotor-Gene 3000 (Corbett Life Science, Sydney, Australia) using the SYBR Green PCR Master Mix (Invitrogen). All the samples were diluted 1/100 with water and 5 μL of these dilutions were used for each SYBR Green PCR reaction containing 10 μL SYBR Green PCR Master Mix, 10 μM of each primer, and deionised water. The reactions were incubated for 10 minutes at 95°C followed by 40 cycles with 15 seconds denaturation at 95°C, 20 seconds annealing at 60°C, and 30 seconds extension at 72°C. Primer sequences are available on request.

### Quantitative real-time PCR data expression and analysis

After completion of the PCR amplification, the data were analysed with the Rotor-Gene software (Corbett Life Science) and Microsoft Excel. In order to quantify the mRNA expression levels, the housekeeping gene *HPRT *was used as a relative standard. All the samples were tested in triplicate. By means of this strategy, we achieved a relative PCR kinetic of the standard and the sample. For all qPCR analyses, RNA from three independent replicates for both wildtype and *Nfib *mutants were interrogated. Statistical analyses were performed using a two-tailed unpaired *t-*test. Error bars represent the standard error of the mean.

### Diffusion-weighted magnetic resonance imaging and tractography

Following perfusion fixation and phosphate-buffered saline washing, diffusion-weighted images were acquired with the samples immersed in Fomblin Y-LVAC fluid (Solvay Solexis, Italy), using a 16.4 Tesla Bruker scanner and a 10 mm quadrature birdcage coil. A three-dimensional diffusion-weighted spin-echo sequence was acquired using a repetition time of 400 ms, an echo time of 22.8 ms and an imaging resolution of 0.08 × 0.08 × 0.08 mm with a signal average of 1. Each dataset was composed of two *B*_o _and thirty direction diffusion-weighted images (*b *value of 5,000 s/mm^2^, δ/Δ = 2.5/14 ms). Reconstruction and tractography were performed with Diffusion Toolkit [51] according to high angular resolution diffusion (HARDI) and Q-ball models [52]. Tractography limits were set at fractional anisotropy values greater than 0.1 and a turning angle ≤ 45°. Hippocampal commissure tractography was performed using hand-drawn regions-of-interest on colour-coded fractional anisotropy maps in TrackVis [53].

## Abbreviations

CC: corpus callosum; DCC: Deleted in colorectal cancer; DTMRI: diffusion tensor magnetic resonance imaging; E: embryonic day; GFAP: glial fibrillary acidic protein; GLAST: astrocyte-specific glutamate transporter; Npn1: neuropilin 1; PFA: paraformaldehyde; qPCR: quantitative real-time PCR.

## Competing interests

The authors declare that they have no competing interests.

## Authors' contributions

MP and LJR conceived the study, evaluated the findings, prepared the figures and the manuscript. RMG generated the *Nfib *mutant strain. MP, RXM, CL, EL, GB, SM, NS and NDK carried out all the experimental procedures.

## Supplementary Material

Additional file 1 **Figure S1 - Co-expression of NFIB and β-galactosidase in *Nfib *heterozygous mice**. (A-I) Confocal image of an E18 *Nfib *heterozygote brain demonstrating expression of (β-galactosidase (β-gal, A, D, G) and NFIB (B, E, H) at the cortical midline. (A-C) Low power image of the cortical midline, demonstrating extensive co-expression of β-gal and NFIB (C). (D-F) Higher magnification view of the glial wedge (GW), demonstrating that the majority of cells in this region co-express NFIB and β-gal, in particular those cells located at the ventricular surface (arrowhead in F). (G-I) Higher magnification view of the indusium griseum glia (IGG), showing that these cells express both NFIB and β-gal (arrows in I). Scale bar: A-C 200 μm; D-I 100 μm. Click here for file

Additional file 2 **Figure S2 - Reduction of tenascin C expression in the absence of *Nfib***. Coronal sections of wildtype (A, C) and *Nfib*-deficient (B, D) brains. Expression of the glial marker tenascin C was reduced at the cortical midline of mice lacking *Nfib *at both E15 (B) and E16 (D). Scale bar: A, B 280 μm; C, D 250 μm. Click here for file

Additional file 3 **Figure S3 - Expression of NFIB and Satb2 in the neocortex**. Confocal image of an E18 wildtype brain. (A) DAPI nuclear staining; the boxed region indicates the position of panels B-D. Satb2, a marker for callosally projecting axons, was expressed predominantly in upper layers of the cortical plate (B). NFIB expression was highest in the deeper cortical layers, but was also seen in layers II/III (C). Most cells within the cortical plate did not co-express NFIB and Satb2 (D), although some overlap was evident in layers II/III. Scale bar: A 500 μm; B-D 100 μm. Click here for file
