# Supplementary figures and images for: Multiple non-cell-autonomous defects underlie neocortical callosal dysgenesis in Nfib-deficient mice
Source: Neural Dev. 2009 Dec 4;4:43. doi: 10.1186/1749-8104-4-43 (PMC2802587; doi:10.1186/1749-8104-4-43)

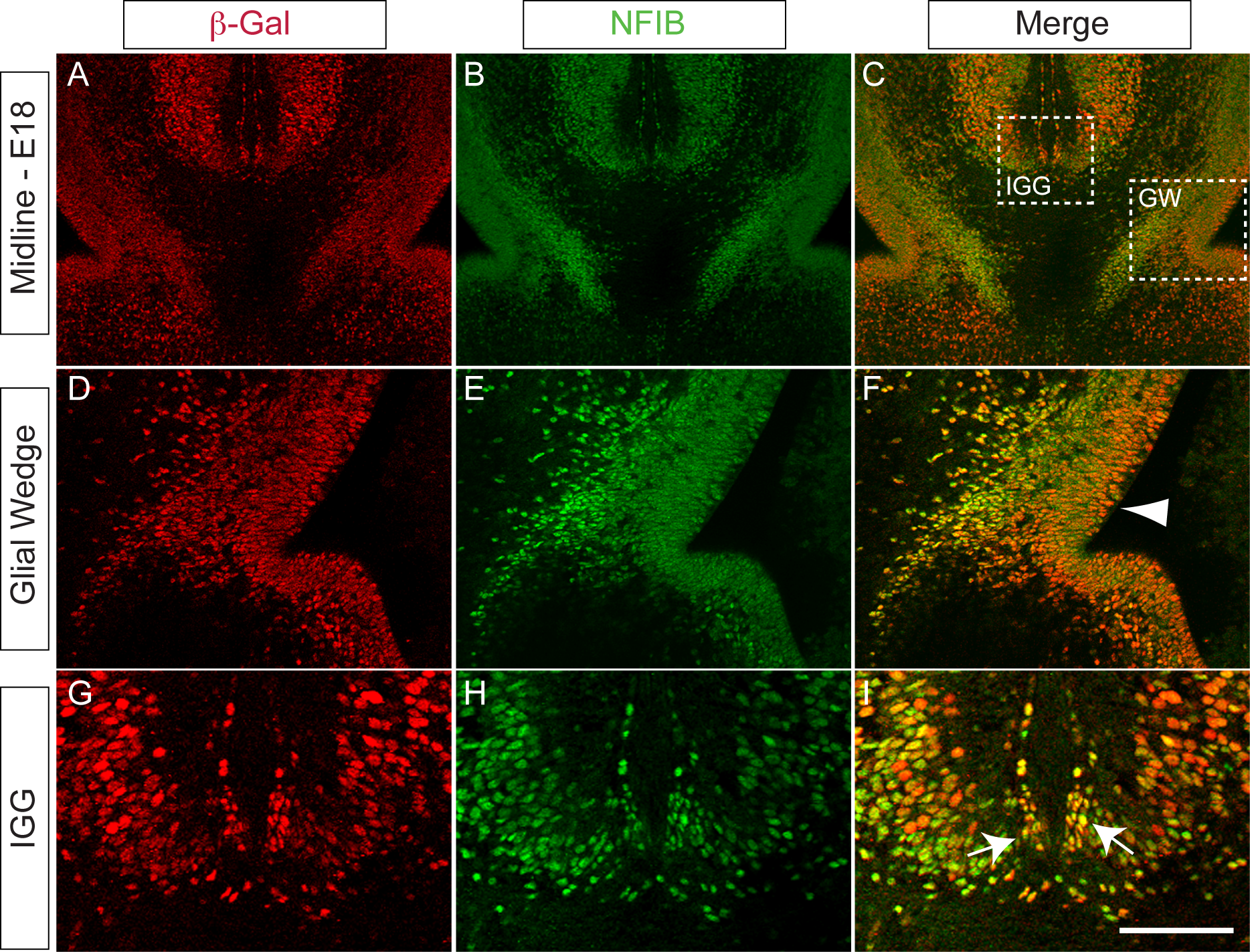

Supplement: Additional file 1 — Figure S1 - Co-expression of NFIB and β-galactosidase in Nfib heterozygous mice. (A-I) Confocal image of an E18 Nfib heterozygote brain demonstrating expression of (β-galactosidase (β-gal, A, D, G) and NFIB (B, E, H) at the cortical midline. (A-C) Low power image of the cortical midline, demonstrating extensive co-expression of β-gal and NFIB (C). (D-F) Higher magnification view of the glial wedge (GW), demonstrating that the majority of cells in this region co-express NFIB and β-gal, in particular those cells located at the ventricular surface (arrowhead in F). (G-I) Higher magnification view of the indusium griseum glia (IGG), showing that these cells express both NFIB and β-gal (arrows in I). Scale bar: A-C 200 μm; D-I 100 μm. [file 1749-8104-4-43-S1.TIFF]

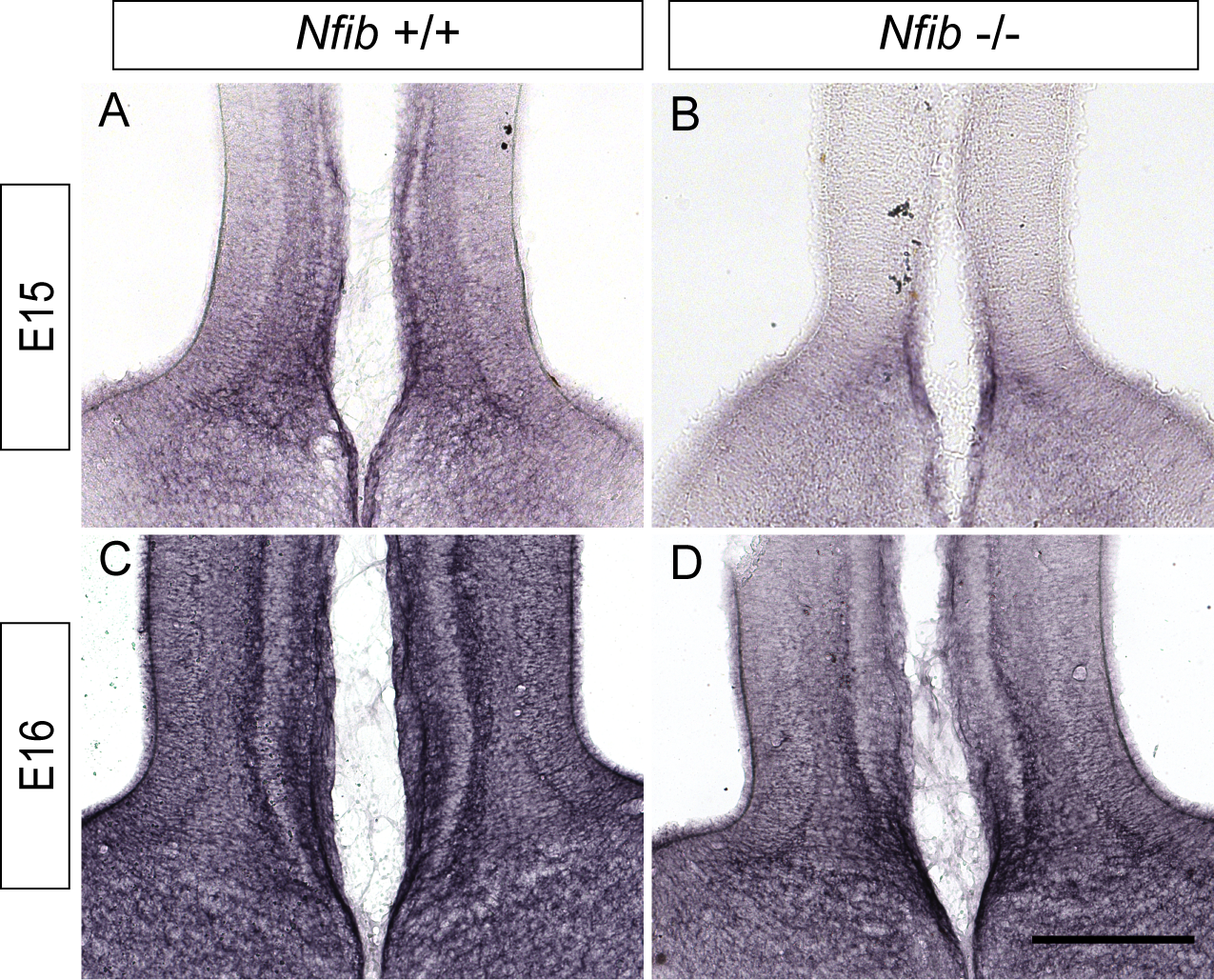

Supplement: Additional file 2 — Figure S2 - Reduction of tenascin C expression in the absence of Nfib. Coronal sections of wildtype (A, C) and Nfib-deficient (B, D) brains. Expression of the glial marker tenascin C was reduced at the cortical midline of mice lacking Nfib at both E15 (B) and E16 (D). Scale bar: A, B 280 μm; C, D 250 μm. [file 1749-8104-4-43-S2.TIFF]

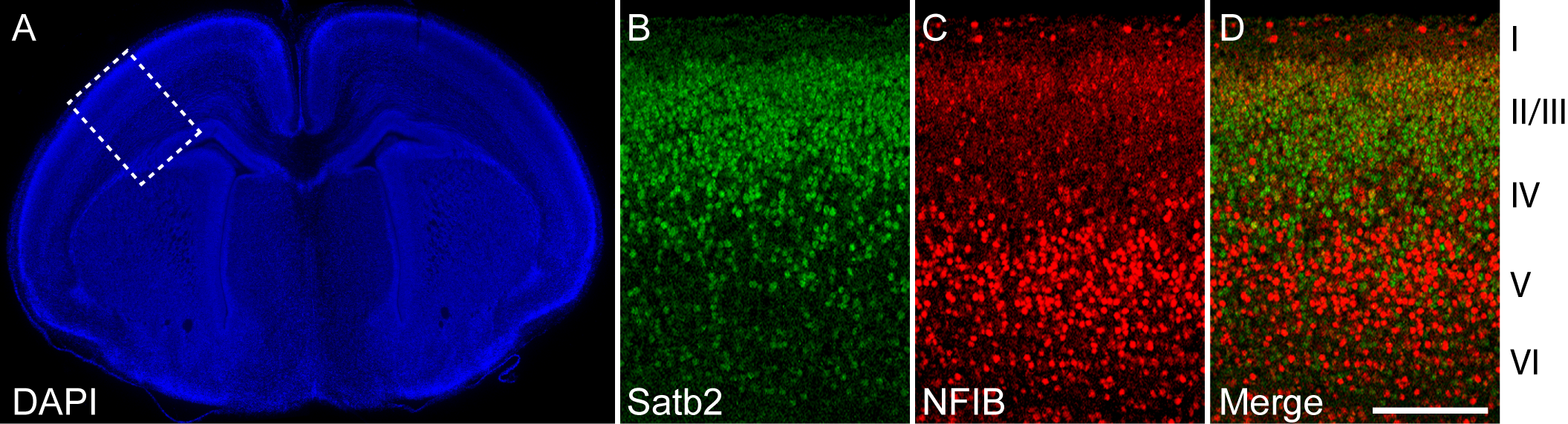

Supplement: Additional file 3 — Figure S3 - Expression of NFIB and Satb2 in the neocortex. Confocal image of an E18 wildtype brain. (A) DAPI nuclear staining; the boxed region indicates the position of panels B-D. Satb2, a marker for callosally projecting axons, was expressed predominantly in upper layers of the cortical plate (B). NFIB expression was highest in the deeper cortical layers, but was also seen in layers II/III (C). Most cells within the cortical plate did not co-express NFIB and Satb2 (D), although some overlap was evident in layers II/III. Scale bar: A 500 μm; B-D 100 μm. [file 1749-8104-4-43-S3.TIFF]
